# Flood risk perception, disaster preparedness and response in flood-prone urban communities of Rivers State

**DOI:** 10.4102/jamba.v14i1.1303

**Published:** 2022-09-29

**Authors:** Zelda A. Elum, Olanrewaju Lawal

**Affiliations:** 1 Department of Agricultural Economics and Extension, Faculty of Agriculture, University of Port Harcourt, Port Harcourt, Nigeria; 2 Department of Geography and Environmental Management, Faculty of Social Sciences, University of Port Harcourt, Port Harcourt, Nigeria

**Keywords:** urbanisation, climate change, floods, sustainability, risk perception, vulnerability

## Abstract

Rapid urbanisation is contributing to increasing societal vulnerability to disaster. This study aimed at exploring the perception on flood risk and ascertaining the determinants of disaster preparedness among residents in flood-prone urban communities. Descriptive statistics and discriminant regression model were employed on primary data collected from 240 urban households across five communities at risk of flooding in the study area. The results showed that most households had low awareness of flood risk and exhibit low levels of adaptive capacity, having adopted little or no measures to deal with disaster floods. Also, awareness of flood risk was observed to discriminate the most between the two groups of adopters and nonadopters of flood preventive and management measures (proxy for disaster preparedness), followed by flood risk perception, age, location and household size.

**Contribution:**

The study suggests an integrated approach (a combination of preventive, protective and control measures) by all stakeholders, including government and other relevant bodies, increasing public awareness of flood risk and its attending effects for greater responsiveness, supporting communities in regular clearing of drainage areas and strictly regulating the construction of buildings, particularly in flood prone areas.

## Introduction

Urbanisation is a growth that defines increasing human activities which have implications for the economic, social, political and physical geography of an area (Caparros-Midwood, Barr & Dawson 2015). Nigeria’s increasing rate of urbanisation has been linked to environmental degradation (Duh et al. 2008). Rapid urbanisation aggravates the challenges already posed by climate change, increasing vulnerability to climate change impacts. Undoubtedly, urbanisation in developing countries is often accompanied by increasing environmental risks and scarcity of resources. Some of the environmental risks magnified by urbanisation include flooding. Its increasing occurrence across the globe has been linked to climate change (Abass et al. 2022). Nigeria in particular is notably identified as vulnerable to climate change–related disasters such as floods, epidemics and droughts on account of the myriads of socio-economic constraints confronting the region and its inhabitants.

Vulnerability describes the susceptability of people or places to damaging impacts arising from exposure to hazard events. In the context of flooding hazards, vulnerability describes how predisposed people or systems are to experiencing floods impacts differently given the variablity in their characteristic features. Vulnerability is influenced by physical, social, economic and environmental factors (Douglas et al. 2008; Lawal & Arokoyu 2015; Tingsanchali 2012), and identiying these factors is a vital pathway to adopting appropriate strategies for coping or mitigating the impacts. Understanding the dynamics of hazards, exposure and vulnerability of communities is important for building their resilience (Etinay, Egbu & Murray 2018). This is because exposure (a component of vulnerability) to hazards like floods is determined by the probability of occurrence of the hazards (e.g. flood risk) and the sensitivity of the people or systems to the impact of the hazard (Brooks 2003), which is dependent on their adaptive capacity and which consequently influences their resilience.

According to Lawal and Umeuduji (2017), flood is the most reoccurring natural disaster in Nigeria, resulting from gradual build-up of rainwater on saturated ground or spontaneously in areas with inadequate storm water management. Flooding is a natural characteristic of rivers where high flow of water overflows the natural banks (Baten, Arcos González & Delgado 2018). However, it is one of the major climate change impacts that have caused people to be displaced from their homes, sometimes causing people to migrate from rural or agricultural communities to urban or nonagricultural settlements (Barrios, Bertinelli & Strobl 2006; De Brauw, Mueller & Lee 2014). For example, the severe floods of 2012 that occurred in many parts of Nigeria including Rivers State left many homes, communities and businesses destroyed (Akukwe, Krhoda & Oluoko-Odingo 2018). As it has been observed in Nigeria and specifically Rivers State, more often than not, the cause and magnitude of floods is attributed to poor physical planning, poor living habits of people (e.g. dumping of refuse in drainage areas and putting up shanties without care on water channels) and disregard for city plans, building codes, environmental rules and regulations in affected communities (Aderogba 2012). Specifically, city expansion due to increasing population in Port Harcourt has been perceivably marred with challenges, resulting in informal settlements and the building of houses on unapproved sites and on flood plains, all in a bid to have cheap accommodation for the growing urban population (Obinna, Owei & Mark 2010). It is on this background that the study sets out to examine vulnerability of urban residents to floods, their awareness and perception of flood disaster risk and to investigate the factors influencing the disaster preparedness and response among the urban dwellers.

Different methodological approaches have been explored in understanding what influences or informs an individual’s risk perception and action. In some studies, the signal detection theory (a visual perception theory) has been applied on the assumption that the detection or recognition of a stimulus or event depends on the stimulus intensity and the physical and psychological state of the individual, which affects the individual’s ability to discriminate more intense stimulus (or dangerous situations) from the less intense or less threatening (Eiser et al. 2012). The physical and psychological state of the individual is itself shaped by numerous factors that can be broadly classified as socio-economic, environmental, cultural and political factors. For instance, previous or direct experience of flood events is assumed to influence one’s risk perception and, in turn, preparedness action, either by aiding the response phase in an organised manner or by causing a low level of personal preparedness, particularly if there is greater reliance on publicly built structures and flood defences (Cologna, Bark & Paavola 2017). Risk perception has been studied with different variables or the same variables operationalised in different contexts (Netzel et al. 2021). Nonetheless, it is assumed that perception of disaster risk involves taking a decision (responses) that is informed by the individual’s sensitivity to the risk impact or its severity and the level or amount of information about the risk that is made available to the individual. In other words, risk communication is an important aspect of assessment of risk perception and adoption of risk management strategies. Risk communication is about making people aware of disaster risk and how they can be affected and to take actions towards reducing the impacts well ahead of the occurrence of disaster events (United Nations 2016). Often, risk is communicated to encourage precautionary measures (Netzel et al. 2021) among the stakeholders at risk. The models employed for risk communication are very much dependent on the direction of communication, the roles of the communicator and the receiver and the purpose of the communication (Rollason et al. 2018). Flood risk communication entails identifying flood-prone areas and letting stakeholders know the causes and likelihood of floods occurring as well as the probability of damage (Demeritt & Nobert 2014; Rollason et al. 2018). Effective risk communication creates greater risk awareness that informs how the people percieve them as important issues that need disaster preparedness actions or response for disaster risk reduction (Barquet & Cumiskey 2018).

Disaster risk reduction, in the context of flooding, is acquiring adequate capacity and knowledge to build sustainable infrastructures so as to reduce people’s exposure and vulnerability to flood hazard (Kwak, Muraoka & Asai 2018). There are two broad approaches to disaster risk reduction (Intergovernmental Panel on Climate Change [IPCC] 2007), the top-down (based on institutional responses) and bottom-up (based on local communities capacity to adapt and prepare for disaster). Thus, there is a connection between disaster risk reduction and disaster preparedness. Studies have shown that stakeholders’ adoption and effectiveness of flood risk management strategies is highly dependent on their perception and attitudes towards flood risk (Santoro et al. 2019). Effectiveness in this context is when the benefits of adopting management strategies offset the impacts of the flood in comparison to the outcomes in a nonadoption scenario. Empirically, logistic regression has been used to investigate the relationship between public risk perception of floods and implementation of mitigation measures, using variables such as age, gender, number of children, years living in the same house and highest educational attainment (Netzel et al. 2021). Studies (Baten et al. 2018; Nur & Shrestha 2017; Rakib et al. 2017) have documented that children, women and the elderly are among the most affected during flooding because of their lack of physical capacity to bear flooding impacts. Therefore, a household with a greater number of vulnerable persons that buys insurance to guard against disaster loss or keeps a family evacuation plan could substantially minimise loss and damage (Hoffmann & Muttarak 2017) from flood hazards. In Hoffmann and Muttarak (2017), diverse factors broadly classified into sociodemographic (e.g. education, income level, marital status, children and the aged present in households), structural or geographical (e.g. length of residence, natural environment and hazard risks) and psychosocial factors (e.g. hazard awareness, knowledge and risk perception) have been identified as significant determinants of disaster preparedness behaviour as well as effective responses.

**PLATE 1:**
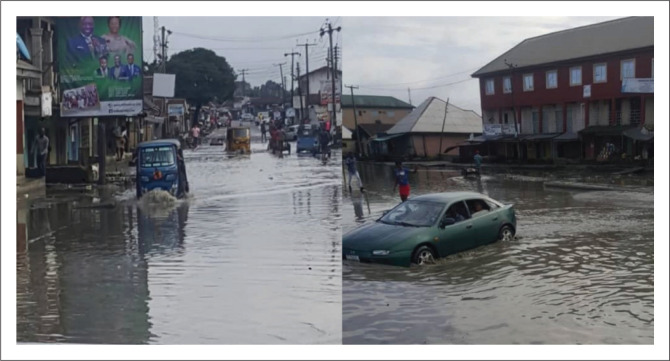
Photo of flooded areas in Port Harcourt metropolis.

In Port Harcourt, some areas are naturally prone to flooding (Plate 1) while others experience flood as a result of unplanned development whereby houses are built on valleys, floodplains and water channels due to necessity and pressure to supply affordable housing for the increasing urban population (Gerald-Ugwu, Egolum & Emoh 2019). By implication, those living in these houses built on floodplains are increasingly vulnerable to flood disaster. Studies on flooding and urbanisation in Port Harcourt have often focused on causes of urban flooding or on the socio-economic and environmental effects. Urban floods can result in significant economic losses directly and indirectly from loss of property and infrastructure, loss of livelihood and poor health (Baten et al. 2018). While the associated hazards are established, there is limited literature on the disaster preparedness of the households for effective response mechanism and how it is shaped by their adaptive capacity. Thus, this study becomes apt, as it is necessary to examine the flood risk perception of urban residents and to identify local factors shaping their preparedness behaviour for effective response to flooding impacts in Rivers State. The study is guided by three research questions: (1) what is the state of awareness and perception of urban households about disaster flood risk? (2) What is the adaptive capacity of the households to cope with floods impact? (3) How do households’ risk awareness, perception and other socio-economic factors relate to their preparedness in dealing with flood event? The study by providing answers to these questions will help stakeholders to design appropriate measures for flood prone areas in Rivers State.

## Materials and methods

**FIGURE 1:**
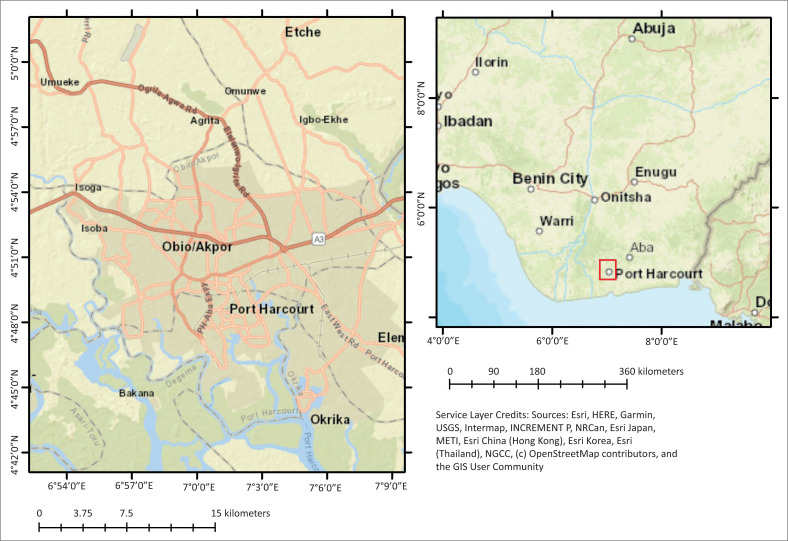
Obio-Akpor local government area and its environs.

The study was done in Rivers State (Figure 1). Located on the Atlantic Coast of southern Nigeria, the state covers an area of 10 575 km^2^ (Federal Republic of Nigeria [FGN] 2011). The state is made up of 23 local government areas (LGAs), with a population of 11.5 million people (FGN 2011). The state capital, Port Harcourt, is popular for its oil and gas industry, and as such, it experiences a high level of commercial activities fuelled by the presence of oil multinationals operating in the state (Obinna et al. 2010). Port Harcourt, by virtue of its status as an oil city, tends to have a high population influx (Potts 2015). Port Harcourt lies within the floodplains of the River Niger (Mmom & Iluyemi 2018). Data was collected through a multistage sampling technique. The first stage involved a purposive selection of Obio-Akpor LGA, which has been identified as highly vulnerable due to its river floodplain settlements and proximity to rivers and creeks (Akukwe & Ogbodo 2015). In the second stage, five flood-prone urban communities were selected.

In the third stage, using the total number of households in the communities as sampling frame, a 2% proportionate sampling was employed on a systematic random sampling of a total of 240 households. This sample size (2% of total population) was chosen due to monetary constraints. However, it is above the 100 minimum sample size number allowable for meaningful research. More so, the sample size given the population size when computed according to Yamane (1967 as cited in Tangonyire and Akuriba, 2021) sample size formula, falls between an acceptable ± 5% to ± 7.5% margin of error or uncertainty (Conroy 2018). The distribution of sample drawn across the communities is presented in Table 1.

**TABLE 1:** Sample selection plan.

Community	Sampling frame(number of households)	Sample size at 2% proportionate sampling
Nkpolu	700	14
Rumuigbo	1300	26
Rumuekpirikom	3800	76
Rumueme	3500	70
Rumukalagbor	2700	54

**Total**	**12 000**	**240**

The data were collated for the variables listed in Table 2. These were explored and analysed with descriptive statistics and discriminant analysis respectively. Discriminant analysis builds a predictive model for group membership. In this study, it was used to predict whether a household is disaster prepared or not. Discriminant analysis is a parametric analysis that helps to determine which of the independent variables will discriminate between the groups that makes up the dependent variable. The variables’ contributions to predicting of group membership are determined by the size of their standardised regression coefficients (Ramayah et al. 2010). The model specification is as follows:


$$Y_{ij}=a+b_{1}X_{ik}+b_{i}X_{2k}+\ldots .+b_{n}X_{nk}$$ [Eqn 1]

**TABLE 2:** Definition of variables used in analysis.

Variables	Description
Age	Age of respondent measured in years
Household size	Number of persons living with respondent
Households with children less than 5 years	Number children less than 5 years
Households with dependants older than 60 years	Number of dependants older than 60 years
Gender	Gender of respondent (dummy: male = 1 otherwise 0)
Years of residency in the area	Number of years living in the area
Education	Categorical and ordinal; 0 = none, 1 = primary, 2 = secondary, 3 = tertiary
Disaster preparedness	Dummy; if any flood preventive or mitigation actions have been taken = 1 otherwise 0
Experience of past floods	Dummy variable; 0 if never affected by flood, otherwise 1
Risk awareness	Dummy; 1 if have information on flooding risk otherwise 0
Perception of flood risk	Categorised as low = 1, moderate = 2, high = 3
Perception on climate change causing flood	Respondents’ view of climate change as cause of flooding; dummy. Agree = 1 otherwise 0
Willingness to purchase insurance	Dummy: Yes = 1 otherwise 0
Perceived level of vulnerability to flood	Categorised: low = 1, moderate = 2, high = 3

Where *Y_ij_* is the discriminate *Y* score for object *k’s* discriminant function *j*. *A* is the intercept, *b*_*i*_ is the coefficients for the independent variable *i* and *X_j_* is the independent variable for object *k*. Specifically, the stepwise discriminant analysis is used.

The cutting score for classifying the observations was calculated as in Ramayah et al. (2010):


$$Z_{cs}=\frac{N_{A}Z_{B}+N_{B}Z_{A}}{N_{A}+N_{B}}$$ [Eqn 2]

Where *Z_cs_* is optimal cutting score, *N_A_* and *Z_A_* are the number of observations and centroid for group *A*, respectively, while *N_B_* and *Z_B_* are the number of observations and centroid for group *B* respectively. The decision rule is that observations with discriminant scores less than the cutting score are classified into group (0), while those with scores that are higher are classified together as belonging to group (1). At each step in the stepwise discriminant analysis, the variable that minimises the overall Wilk’s Lambda is entered. The test of equality of group means shows if there are statistically significant group differences with respect to the independent variables. Also, because the model contained four dummy variables, a hierarchical discriminant analysis was done to know the effect of the dummy variables as they cannot be interpreted like other variables in the linear regression model. Therefore, a discriminant analysis was first done without the dummy variables and then with the variables. Thereafter, the difference in the canonical correlation was computed, and this indicates a joint explanatory effect of the dummy variables as a set. In addition, the structure matrix correlations are used in ranking the variables in order of importance, as it shows the correlation of each explanatory variable with the dependent variable, and they are considered more accurate than the standardised coefficients. However, both the standardised and unstandardised coefficients indicate the partial contribution of each significant variable, holding other variables constant.

A household’s vulnerability perception was categorised based on the number of stressors indicated, whereby if a household indicated the presence of 30% or less of the total stressors, it was rated as having low vulnerability to flooding. Those characterised by 40% – 60% of the stressors were grouped as moderately vulnerable, while households that indicated between 70% and 100% of the stressors were perceived to have high level of vulnerability to flood. Likewise, the flood risk perception of the households was categorised into three categories based on the number of flood impact indicators experienced. This assumes that an individual’s perception is shaped by experience, and as such they will act only if a hazard risk had and will make significant impact. Therefore, a household that had experienced 30% or fewer of the possible impacts was classified as having low flood risk perception. Those who had experienced up to 40% – 60% of the listed impacts were rated as having moderate perception, while those who had experienced about 70% – 100% of the impacts were rated as having high flood risk perception. Similarly, based on the number of responses on adoption of precautionary measures, the households were categorised into low adaptive capacity and high adaptive capacity groups, using a cut-off point of five obtained as the midpoint of the 10 presented adaptive measures requiring a yes or no response. Residents who indicated a yes to fewer than or equal to 5 measures were considered a low adaptive capacity group; otherwise, they were considered to have high adaptive capacity.

### Ethical considerations

Ethical approval to conduct this study was obtained from the University of Port Harcourt Research Ethics Committee at its 65th meeting held 02 October 2019 (reference number: UPH/CEREMAD/REC/MM65/033).

## Results and discussion

### Socio-economic characteristics of the urban households

It is observed in Table 3 that the average age of the residents was 38 years, and a slight majority (52.3%) had children who were less than 5 years old in their households. A majority (61%) of the respondents were male and the respondents had on average lived in the communities for 7 years, implying that they may have experienced some of the past and recent incidences of flooding in the area. Also, a majority (98.5%) have had one form of education. Furthermore, 55.3% of the residents were not disaster prepared, as they have not taken any precautionary actions, compared to 44.7% that felt otherwise. Many (77.3%) had experienced floods, yet a majority 74.4% had low to moderate flood risk perception. In contrast to Hoffmann and Muttarak’s (2017) opinion that education and flood experience could trigger a training process that has the potential to increase preparedness levels, it is seen from the descriptive results that a majority of the respondents (69%), despite their literacy and experience of flooding, are not willing to buy insurance, a form of disaster preparedness measure. This could be partly attributed to the observation that a majority (65%, 74% and 60%) of the respondents were not aware of the risks posed by flooding, had low perception of flood risk and did not agree on climate change being a driver of recent occurring floods, respectively. The result is similar to the findings of Akukwe and Ogbodo (2015), in which the highest proportion of their respondents had little awareness of floods, and the result also supports the notion that flooding in Port Harcourt is mostly caused by human factors (Akukwe 2014). Also, a majority (72%) were grouped as having high vulnerability to flooding given the level of stressors they perceived as present in their environment. More so, a higher proportion (75%) of the respondents were presently employed and 25% unemployed (including students and the retired).

**TABLE 3:** Descriptive characteristics of the urban households.

Variables	Total	Frequency	%	Mean	Minimum	Maximum
Age of respondents	198	-	-	37.95	17	80
**Household size**	199	-	-	4.65	1	20
Households with children less than 5 years	199	104	52.3	-	0	12
Households with dependants older than 60 years	198	44	22.2	-	0	4
**Gender**	199	-	-	-	0	1
Male	-	122	61.3	-	-	-
Female	-	77	38.7	-	-	-
Years of residence in the area	198	-	-	6.89	1	42
**Education**	199	-	-	-	0	3
No formal education	-	3	1.5	-	-	-
Primary	-	16	8.0	-	-	-
Secondary	-	72	36.2	-	-	-
Tertiary	-	108	54.3	-	-	-
**Flood risk awareness**	185	-	-	-	0	1
No	-	120	64.86	-	-	-
Yes	-	65	35.14	-	-	-
Source of information on flood	65	-	-	-	0	1
Media (radio, TV and social media)	-	63	96.92	-	-	-
Nonmedia (friends and family)	-	3	4.62	-	-	-
**Disaster preparedness**	199				0	1
No		110	55.3		-	-
Yes		89	44.7		-	-
**Experience of past floods**	199				0	1
No	-	45	22.7		-	-
Yes	-	153	77.3		-	-
**Perception of flood risk**	199	-	-	-	1	3
Low	-	79	39.7	-	-	-
Moderate	-	69	34.7	-	-	-
High	-	51	25.6	-	-	-
**Perception on climate change causing flood**	183	-	-	-	0	1
Agree	-	74	40.4	-	-	-
Disagree	-	109	59.6	-	-	-
**Willingness to purchase insurance**	184	-	-	-	0	1
No	-	126	68.5	-	-	-
Yes	-	58	31.5	-	-	-
**Perceived level of vulnerability to flood**	199				1	3
Low	-	15	7.5	-	-	-
Moderate	-	41	20.6	-	-	-
High	-	143	71.8	-	-	-
**Location**	183	-	-	-	0	1
Floodplains’ settlement		1164	82.83	-	-	-
Elevated areas		35	17.17	-	-	-
**Employment status**	183	-	-	-	0	1
Presently employed	-	149	74.87	-	-	-
Presently unemployed	-	50	25.13	-	-	-

As shown in Table 4, a majority of the responses occurred under those who were not willing to purchase (WTP) insurance, and a majority (86.51%) of them had either secondary or tertiary education. A lower number of those with secondary education (24.14%) were WTP insured just as a much higher proportion of those with tertiary education (74.14%) were WTP insured. The chi-square test was used to test if these differences were real or due to chance variation. Since the chi-square statistic is less than 0.002, it could be concluded that the difference in the respondents’ willingness to purchase insurance is real and not due to chance. However, given that not all respondents had experienced flood in the past, which could have influenced their willingness to purchase insurance, further cross-classification by previous experience of flood was performed. The chi-square significance of those who had not experienced flood and were not WTP insured was 0.132, suggesting but not conclusive of chance variation between nonwillingness to purchase insurance and education. While those who had previous flood experience and WTP insurance had a significance value of 0.001, implying that the relationship observed in the cross-tabulation is real and not a result of chance. Similarly, all symmetric measures were significant and their values greater than 0.3, implying a strong relationship. Therefore, there is need for further education and enlightenment on insurance markets or products to encourage more participation in the insurance market. To further reinforce this result, cross-classification of willingness to pay for insurance, location and flood experience was done. It was observed that a greater proportion of respondents approximately 29%, 29% and 19% from floodplain settlements of Nkpolu, Rumuigbo and Rumuekpirikom, respectively, were more willing to pay for insurance than those in higher-elevation area of Rumukalagbor. More so, a greater share of respondents who had experienced floods and were also more willing to buy insurance came from the floodplain areas of Rumuigbo (37%), Nkpolu (33%) and Rumuekpirikom (20%). An analysis of variance with respect to flood experience across the locations showed two homogeneous subsets, a significant difference between one location (Rumukalabor) a high-elevation area and the other four (Nkpolu, Rumuigbo, Rumueme and Rumuekpirikom) established as flood-prone areas with floodplain settlements (Nwankwoala & Jibril 2019).

**TABLE 4a:** Cross-tabulation of differences in education and location among respondents characterised by willingness to purchase insurance.

Variable	Nonwillingness to purchase insurance (0)		Willingness to purchase insurance (1)	
	*n*	%	*n*	%
**Education**				
No formal education	3	2.38	0	
Primary	14	11.11	1	1.72
Secondary	51	40.48	14	24.14
Tertiary	58	46.03	43	74.14
Total	126	100.00	58	100.00
**Location**				
Nkpolu	33	26.19	17	29.31
Rumuigbo	19	15.08	17	29.31
Rumuekpirikom	29	23.02	11	18.97
Rumueme	23	18.25	2	3.45
Rumukalagbor	22	17.46	11	18.97
Total	126	100.00	58	100.00

Note: Pearson chi-square test = 0.002 for education among respondents characterised by willingness to purchase insurance. Pearson chi-square test = 0.026 for location among respondents characterised by willingness to purchase insurance.

**TABLE 4b:** Cross-tabulation of differences in education among respondents characterised by willingness to purchase insurance.

Variable	Non-willingness to purchase insurance (0)				Willingness to purchase insurance (1)			
	Flood experienced				Flood experienced			
	No (0)		Yes (1)		No (0)		Yes (1)	
	*n*	%	*n*	%	*n*	%	*n*	%
**Education**								
No formal education	-	-	3	2.80	-	-	0	-
Primary	3	16.67	11	10.28	0	-	1	2.17
Secondary	1	5.56	50	46.73	3	25.00	11	23.91
Tertiary	14	77.78	43	40.19	9	75.00	34	73.91
Total	18	-	107	-	12	-	46	-

Note: Flood experienced - Pearson chi-square test, Phi, Cramer’s V and Contingency coefficient = 0.132 for education among respondents characterised by non-willingness to purchase insurance.

Pearson chi-square test = 0.001 for education among respondents characterised by willingness to purchase insurance.

**TABLE 4c:** Cross-tabulation of differences in location among respondents characterised by willingness to purchase insurance.

Variable	Non-willingness to purchase insurance (0)				Willingness to purchase insurance (1)			
	Flood experienced				Flood experienced			
	No (0)		Yes (1)		No (0)		Yes (1)	
	*n*	%	*n*	%	*n*	%	*n*	%
**Location**								
Nkpolu	1	5.56	2	16.67	32	29.91	15	32.61
Rumuigbo	-	-	-	-	18	16.82	17	36.96
Rumuekpirikom	5	27.78	2	16.67	24	22.43	9	19.57
Rumueme	2	11.11	1	8.33	21	19.63	1	2.17
Rumukalagbor	10	55.56	7	58.33	12	11.21	4	8.70
Total	18	-	12	-	107	-	46	-

Note: Flood experienced - Pearson chi-square test, Phi, Cramer’s V and Contingency coefficient = 0.721 for location among respondents characterised by non-willingness to purchase insurance.

Pearson chi-square test = 0.012 for location among respondents characterised by willingness to purchase insurance.

Furthermore, nine local stressors that could influence a community or an individual’s vulnerability to flooding were identified and presented to the respondents to indicate which stressors were perceivably present in their community or had been experienced.

It is seen from the result in Table 5 that poorly constructed or blocked drainage, where available, unplanned building of structures, frequent rains, inadequate drainage systems, poor environmental sanitation monitoring by government, congestion and lack of awareness of government environmental management practices in the community are major factors perceived to be predisposing the communities to flooding. As noted in Appleby-Arnold et al. (2018), when people perceive that government authorities are not working effectively, it creates in them resigned attitudes that can also hamper disaster preparedness. The major impacts of flooding suffered by the residents included cutting off of electricity supply to houses, disruption of household activities and loss of property. It can be deduced from the result that the vulnerability factors characterising the households were majorly structural (drainage issues and unplanned constructions), climatic (excessive rainfall), environmental and public utility–related (inadequate and poorly maintained environment or drainages). The result agreed with Uddin (2018) that poor implementation of urban plans and housing policies leads to the creation of slums and shanties lacking in basic facilities and impacting negatively on human security (e.g. access to food and electricity). Also, as noted by Echendu (2020), poor waste management contributes to flooding occurrence. There is no doubt that flooding will greatly impact areas where drainage structures are deficient, as seen in many urban areas. However, a flood-resilient community can emerge if the aforementioned challenges are addressed, thereby enhancing its ability to mitigate flood impacts and minimise vulnerability (Chong, Kamarudin & Abd Wahid 2018).

**TABLE 5:** Assessment of urban households’ vulnerability and disaster preparedness.

Vulnerability characteristics	Frequency	%	Rank
Inadequate drainage system	169	91.85	5th
Poorly constructed drainage	182	98.91	1st
High frequent rains	173	94.02	3rd
Blocked drainage areas	173	94.02	3rd
Unplanned building of structures	174	94.57	2nd
Poor maintenance of environment by designated government agencies	142	77.20	6th
Congested population in communities	113	61.41	8th
Flooding from small streams whose catchment areas lie within built-up areas	72	39.13	9th
Lack of awareness of government environment management practices within the community	142	77.17	6th
**Experienced impacts of flooding**			
Experience shortage of food during floods	61	38.10	7th
Experience shortage of clean drinking water during flood	40	25.00	9th
Toilet facilities are affected in time of flood	59	37.30	8th
Power (electricity) cut when flood occurs	137	88.40	1st
Loss of property during flood	101	63.10	2nd
Suffered body injuries as result of flood	68	44.16	5th
Loss of a loved one as a result of the flood	17	11.04	10th
Flood disrupted household activities	89	57.79	3rd
Household building was damaged as a result of flood	86	55.84	4th
Experience disruption of income generating business because of flood	77	48.40	6th
**Coping or adaptive measures**			
Use of mechanical water pumps to remove flood water from home	60	42.55	4th
Built temporary plank bridges between houses and across roads to move about during flooding	75	52.08	3rd
Constructed dykes or trenches to divert water away from the house	27	19.29	8th
Relocated to highest parts of community that are more secure from flood	55	39.29	6th
Purchased insurance policy to guard against disaster loss	14	7.57	10th
Constructed drainages around property	106	58.56	1st
Build walls around building to keep out water	98	54.44	2nd
Planted vegetation around building to reduce or prevent erosion	21	11.41	9th
Aware of government disaster management agency to call on in the occurrence of disaster event	77	41.85	5th
Involved in joint communal effort to combat flood	50	28.57	7th
**Adaptive capacity**			
Low adaptive capacity	157	87.71	-
High adaptive capacity	22	12.29	-

In addition, the number of coping or precautionary measures undertaken by the residents to deal with occurring floods was used as a proxy for gauging their adaptive capacity. Frequency analysis of dichotomous responses given on a list of subjective well-being measures presented to the residents to indicate the adaptive measures they had undertaken. As shown in Table 5, Most (88%) of the surveyed households fell into the low adaptive capacity category. These households generally were associated with lower number of adaptive strategies to mitigate the impacts of floods or respond to future flooding.

### Determinants of flood disaster preparedness

The summary of stepwise discriminant analysis showing the variables influencing the disaster preparedness of a household is presented in Table 6. It could be seen from the *F*-statistics that the variables age, flood risk assessment, flood risk perception, household size and location were all statistically significantly different between those who were disaster prepared and those who were not. In addition, the pooled-within group matrices showed that the intercorrelations were low, justifying the use of the independent variables. Four of the five significant variables have positive coefficients, which means they help to discriminate the households that are disaster prepared (they can drive preparedness behaviours), while the age variable with a negative sign helps to predict the households that are not disaster prepared. In other words, households that have higher number of family members, are aware of the risks associated with floods, have higher perception of flood risk and live in low-lying floodplain settlements will have the tendency to be disaster prepared against possible future flooding disaster. However, younger heads of households will have less tendency to be disaster prepared. In addition, it can also be inferred that flood risk awareness discriminates the most followed by flood risk perception age, location and household size. The result agrees with the literature that location and risk perception can influence disaster preparedness behaviours (Hashim et al. 2021; Najafi et al. 2015).

**TABLE 6:** Summary of measures in the discriminant analysis (*n* = 199).

Independent variables	Unstandardised coefficients	Standardised coefficients	Discriminate loadings (rank)	*F*-statistics
Age (*x*_1_)	−0.06	−0.61	−0.38 (3rd)	68.01*
Risk awareness (*x*_2_)	1.70	0.66	0.62 (1st)	74.61*
Perception on flood risk (*x*_3_)	0.66	0.46	0.44 (2nd)	90.92*
Household size (*x*_4_)	0.09	0.26	0.21 (5th)	54.88*
Location (*x*_5_)	0.79	0.28	0.37 (4th)	46.08*

* , *p* < 0.001.

Intercept, –0.77; Group centroid (0), –1.104; Group centroid (1), 1.193; Wilk’s Lambda, 0.43*; Test of equality of variance (Box’s M test), 176.90*; Canonical correlation (with dummies), 0.76; Canonical correlation (without dummies), 0.49; Effect of dummies taken as a set or whole, 0.27; Overall hit ratio, 87.5%.

The discriminant function (equation) can be obtained from the unstandardised coefficients shown in Table 6 and in this case, it is:


$$D=X=[1\text{ Age Awareness Floodperception Householdsize Location}\left[\begin{array}{c} -0.77\\ -0.06\\ 1.70\\ 0.66\\ 0.09\\ 0.79 \end{array}\right]+e$$ [Eqn 3]

Furthermore, the group centroids (mean of canonical variables) are different for each group. Disaster nonprepared group had a mean of −1.104 while the disaster prepared group was 1.193. The canonical correlation measures the strength of relationship. With a canonical correlation of 0.76, it can be inferred that 58% (square of the correlation) of the variance in the dependent variable is accounted for by this model, and about 42% is unexplained. As indicated by the Wilk’s lambda (Table 6), the discriminant function is better than having chance separate the two groups. The implication of the results is that government and relevant agencies should create more awareness on impending or potential flood disaster and promote public sensitisation on the dangers associated with flooding and the need to prevent or minimise it, especially in flood-prone areas. Although the Box’s M significant value was an indication of violation of the assumption of equality of covariance matrices, it was not considered a serious problem as the sample was a large one.

**FIGURE 2:**
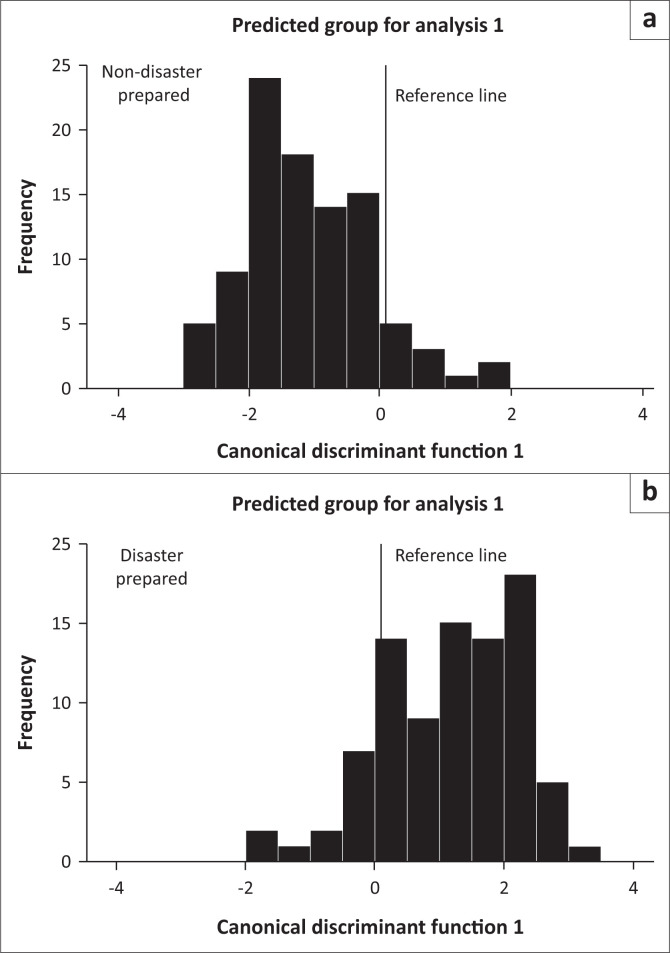
Distribution of discriminant scores for disaster prepared and nonprepared households.

The result in Table 7 showed that households that were not disaster prepared were more accurately predicted (90.6%) than those that were prepared (84.1%). Generally, on the average, 87.5% (hit ratio) of the original group cases were correctly classified. This implies that overall, in three out of four times, the model is correct. The histogram in Figure 2 shows the distribution of the discriminant function scores for each group. It shows how well the function discriminates by the overlap and nonoverlap of the graphs. The cutting score was computed as follows:


$$\begin{array}{l} Z_{cs}=\frac{96(1.193)+88(-1.104)}{96+88}\\ Z_{cs}=\frac{114.528-97.142}{184}=0.09 \end{array}$$ [Eqn 4]

**TABLE 7:** Classification results.

Disaster preparedness (DP)	Predicted group membership					
	0		1		Total	
	*n*	%	*n*	%	*n*	%
Original						
0	87	90.6	9	9.4	96	100
1	14	15.9	77	84.1	88	100
Cross-validated						
0	86	89.6	10	10.4	96	100
1	17	19.3	71	80.7	88	100

NB, Numbers in parenthesis indicate row percentages.

Thus, observations or cases with discriminate score lower than 0.09 are grouped together as non–disaster prepared (coded 0), while cases with scores higher than 0.09 are classified under disaster prepared (coded 1).

## Conclusion

The study examined the vulnerability, flood risk perception and disaster preparedness of urban households in flood-prone areas, as it becomes increasingly important to build resilience to the impacts of climate change-related flood. It also determined the factors that propels a household to be disaster prepared or otherwise. Descriptive statistics and stepwise discriminant analysis were employed on collected data. Results showed that most of the households were unaware of the risks associated with floods and those who were aware became informed through mainstream media. A majority of the participants lived in low-lying floodplain settlements and were employed; a majority had low-medium perception of risk and showed low readiness to deal with a flooding disaster. It was also observed that more of the residents in floodplain settlements experienced flooding and were more willing to buy insurance than those living in elevated areas. Furthermore, given the extent to which they had taken up coping and/or adaptive measures, a majority of the households were classified as having low levels of adaptive capacity. Statistical significance of mean differences was observed for all five predictors (age, flood risk awareness, risk perception, location and household size) out of 15 predictors regressed on the dependent variable. It was shown that the five predictors accounted for 58% of the variation between the two groups variability, while the hit ratio indicated that overall 87.5% of the cases were correctly classified. Important implications arising from the study are that tackling the challenges (e.g. poorly constructed drainage and unplanned building of structures) increasing the vulnerability of the people or area to flooding will enhance the adaptive capacity of the households. Also, creating awareness on disaster risk helps to form the individuals’ perception of the risk and, in turn, their disaster preparedness behaviours and subsequently building resilience. The study supports the need to mitigate flood disaster impacts on households and the environment. It suggests an integrated approach that includes protective, preventive and control measures by all stakeholders, including government and relevant bodies, increasing public sensitisation of flood risk and its attending effects for greater awareness, supporting communities in regular clearing of drainage areas and checking unplanned construction of buildings, particularly in flood-prone areas.
